# Gut-brain axis and vascular dementia: a review on mechanisms and Chinese herbal medicine therapeutics

**DOI:** 10.3389/fmicb.2025.1564928

**Published:** 2025-05-14

**Authors:** Dexiu Wang, Huiying Yao, Luoqi Wang, Bowen Lu, Wenkai Liu, Jinghan Li, Yujin Gong, Yuhao Cai, Yuehan Li, Xin Cai, Rui Zhang

**Affiliations:** ^1^School of Basic Medical Sciences, Shandong Second Medical University, Weifang, China; ^2^School of Clinical Medicine, Shandong Second Medical University, Weifang, China; ^3^Weifang Hospital of Traditional Chinese Medicine, Shandong Second Medical University, Weifang, China

**Keywords:** vascular dementia, gut-brain axis, intestinal flora, Chinese herbal medicine, pathogenesis

## Abstract

Vascular dementia (VD), the second most prevalent form of dementia among the elderly population, is a cerebrovascular disorder characterized primarily by cognitive impairment. Emerging evidence has revealed that intestinal flora dysbiosis may be implicated not only in gastrointestinal (GI) pathologies but also in central nervous system (CNS) disorders, including VD. The gut-brain axis (GBA) serves as a critical bidirectional pathway through which intestinal flora influences brain physiology and function. Notably, accumulating studies have demonstrated the therapeutic potential of Chinese herbal medicine (CHM) in VD management via modulation of gut microbial composition. This review synthesizes current understanding of the VD- intestinal flora relationship mediated by the GBA, while systematically evaluating evidence for CHM interventions that ameliorate VD through intestinal flora regulation. These insights may offer novel perspectives and methodological approaches for both fundamental research and clinical management of VD.

## 1 Introduction

Vascular dementia (VD) is a progressive cognitive disorder caused by chronic cerebral ischemia, recognized as the second most common form of dementia after Alzheimer’s disease (AD). Clinically, VD is characterized by cognitive decline, memory impairment, executive dysfunction, reduced processing speed, and impaired activities of daily living, frequently accompanied by mood disturbances and personality changes (Smith, 2017). Global demographic trends indicate a rapidly aging population, driven by socioeconomic development, cultural advancements, and improved healthcare. Like AD, the prevalence of VD increases significantly with age, contributing to the growing burden of dementia worldwide. Epidemiological data reveal that VD accounts for approximately 15–20% of dementia cases in North America and Europe, while estimates in Asia and developing countries are notably higher, reaching around 30% (Yu and Wang, 2020). Therefore, a deeper comprehension of VD’s underlying mechanisms is essential. Furthermore, the development of novel therapeutic approaches to slow disease progression has become a critical priority in dementia research.

The complex bidirectional interactions between the enteric nervous system (ENS) and central nervous system (CNS) form the gut-brain axis (GBA), which facilitates communication between gut microbiota and the brain. Several researchers have found that patients with neurological disorders (e.g., AD) exhibit significant changes in the composition and structure of their gut flora, characterized by an increase in pathogenic bacteria and a decline in beneficial species. What’s more, studies have shown that when an inflammatory response occurs in the gut environment, inflammatory factors in the brain are also elevated. Therefore, it’s not difficult for us to surmise that the intestines are connected to the brain and that the intestinal flora plays a critical role in brain-to-gut communication. Both preclinical and clinical studies on VD have shown that intestinal flora is involved in mental conditions such as cognitive abilities and mood changes in the host through GBA (Kesika et al., 2021). Intriguingly, with the in-depth study of Chinese herbal medicine (CHM), researchers have discovered that a variety of CHM, ranging from herbal active ingredients to herbal compounds, can mitigate damage in neurological disorders by regulating intestinal flora. Given these findings, we deliberate the implications of improving disturbed intestinal flora while and proposing CHM-based interventions as a promising strategy for VD prevention and treatment.

## 2 The pathogenesis and CHM treatments of VD

### 2.1 Oxidative stress response

Compared to other organs, the brain exhibits lower levels of antioxidant activity and fewer endogenous protective mechanisms, making it particularly vulnerable to oxidative injury. During events such as ischemia, hypoxia, or reperfusion injury, both the biological oxidative function and morphology of mitochondria become abnormal, causing a significant increase in reactive oxygen species (ROS) that overwhelms the brain’s antioxidant defenses. When the levels of ROS exceed a specific threshold, they can cause increased resistance to blood flow, a reduction in vasodilation and immune response, and heightened apoptosis, ultimately culminating in neuronal damage due to oxidative stress. Furthermore, ROS disrupt endothelial nitric oxide signaling, exacerbating cerebrovascular endothelial dysfunction and compromising blood–brain barrier (BBB) integrity (Carvalho and Moreira, 2018). Hence, inhibiting oxidative stress response represents one of the primary methods for preventing and treating VD.

As a bioactive CHM compound, puerarin has been shown to improve vascular endothelial cell function and significantly mitigate oxidative stress in neurons of VD rats primarily through downregulation of pro-apoptotic Bax protein and reduction of the Bax/Bcl-2 ratio (Qi et al., 2023). Additionally, Shenmayizhi decoction (SMYZD) demonstrates significant antioxidant effects in vascular cognitive impairment (VCI), evidenced by decreased malondialdehyde (MDA) levels and increased activities of glutathione (GSH), glutathione peroxidase (GSH-Px), and superoxide dismutase (SOD)(Sun et al., 2021). These findings collectively support that CHM can improve VD by enhancing antioxidant defenses.

### 2.2 BBB damage

Cerebral hypoperfusion and consequent hypoxia predominantly target two critical cellular components of the neurovascular unit: cerebrovascular endothelial cells and astrocytes. These structural and functional compromises to the BBB result in increased vascular permeability, precipitating the development of vasogenic cerebral edema. Rajeev et al. observed structural alterations in the BBB within a hypoperfusion rat model, noting a significant reduction in the expression levels of tight junction proteins ZO-1, occludin, and claudin-5 (Rajeev et al., 2022). This damage facilitates the entry of certain harmful substances into the brain parenchyma, potentially causing irreversible brain damage. Consequently, therapeutic strategies targeting BBB restoration may hold substantial promise in mitigating the progression of VD.

Emerging evidence suggests that traditional herbal formulations may offer neuroprotection. By administering Sanhua Tang (SHT) via gavage, the ultrastructural damage to BBB in rats with ischemic stroke (IS) was alleviated, indicating its potential as a BBB-stabilizing agent (Shan et al., 2024). Similarly, Zhang et al. reported that Pushen capsule ameliorated BBB dysfunction in IS rats, potentially via modulation of the cAMP signaling pathway (Zhang et al., 2023). However, current conclusions are largely confined to animal experiments, and these preclinical findings remain to be substantiated by rigorous clinical investigations.

### 2.3 Excitotoxic effects

Excitotoxicity represents one of the earliest identified and most extensively studied pathophysiological mechanisms in cerebral ischemic injury (Radak et al., 2017). Under conditions of chronic ischemia, disruption of energy synthesis and metabolic homeostasis leads to excessive release of excitatory amino acids (EAAs), accompanied by impaired reuptake mechanisms. Consequently, glutamate accumulates rapidly within the synaptic cleft (Traynelis et al., 2010). Subsequent overactivation of N-methyl-D-aspartate (NMDA) receptors by glutamate induces a massive influx of calcium ions and sustained neuronal depolarization (Akgül and McBain, 2016). The resultant intracellular calcium overload in ischemic brain regions initiates deleterious cascades, including oxidative stress and mitochondrial dysfunction, ultimately culminating in neuronal apoptosis and necrosis. These processes not only exacerbate BBB disruption but also contribute significantly to cognitive impairment and the development of VD (Tu and Kuo, 2015).

Studies in recent years indicate that pharmacological interventions targeting excitotoxicity could provide a promising therapeutic approach for VD. Tian et al. demonstrated Shenzhi Jiannao (SZJN) prescription significantly attenuates glutamate-induced apoptosis in PC12 cells, potentially via regulation of its key targets, Caspase-3, Bax, and Bcl-2 (Tian D. et al., 2022).

### 2.4 Neuroinflammatory response

Cerebral hypoxia-ischemia triggers a robust neuroinflammatory cascade. Following cerebral ischemic injury, damaged neurons release excitatory neurotransmitters and ROS, which rapidly activate resident microglia and recruit peripheral immune cells. These activated immune cells subsequently secrete pro-inflammatory cytokines, including TNF-α and IL-6, amplifying the neuroinflammatory milieu. Concurrently, ischemic stress induces astrocytic swelling and rupture, further activating inflammatory pathways and promoting the release of additional mediators such as IL-8 (Jurcau and Simion, 2021). The resulting inflammatory mediators and cellular exudates infiltrate the neuronal parenchyma, inducing further neuronal damage and triggering a secondary inflammatory response. This pathological cascade ultimately leads to gray matter atrophy and synaptic dysfunction, manifesting as progressive cognitive decline (Tian Z. et al., 2022). Given this pathophysiology, anti-inflammatory strategies are some of the main measures to treat VD.

Li et al. reported that intranasal administration of icariin significantly attenuates hippocampal levels of IL-1β and TNF-α in a rat model of VD, concomitant with improved cognitive performance (Li et al., 2024). Additionally, baicalein has been shown to suppress microglial activation, thereby reducing hippocampal pro-inflammatory cytokine expression and ameliorating cognitive deficits (Song et al., 2024). Notably, these anti-inflammatory principles are now being translated into clinical practice. Liu and colleagues have demonstrated that Shenma Yizhi prescription, when combined with citalopram, significantly reduces serum inflammatory markers and oxidative stress, which will improve cognitive function of patients with cerebral infarction combined with cognitive dysfunction (Liu S. et al., 2021). With accumulating evidence supporting the anti-inflammatory effects of CHM in VD treatment, its application prospects in clinical treatment are expected to become more extensive.

### 2.5 Genetic mechanisms

The genetic mechanisms underlying VD remain inadequately understood; however, the apolipoprotein Eε4 (Apo E4) gene and the NOTCH3 gene are recognized as significant contributors to VD. Studies indicate that individuals carrying the Apo E4 allele have an increased risk of developing VD (Davidson et al., 2006). Similarly, individuals with mutations in the NOTCH3 gene also exhibit a heightened risk of developing VD (Cho et al., 2022). Furthermore, pathogenic mutations in this gene are associated with the development of hereditary subcortical vascular dementia (HSD), which typically presents with early onset and a higher frequency of cognitive impairment among affected individuals with a family history of the condition (Guo et al., 2021). However, there are currently very few cases of treating VD through genetic mechanisms in CHM, which is expected to become a future research direction.

**Figure 1:**
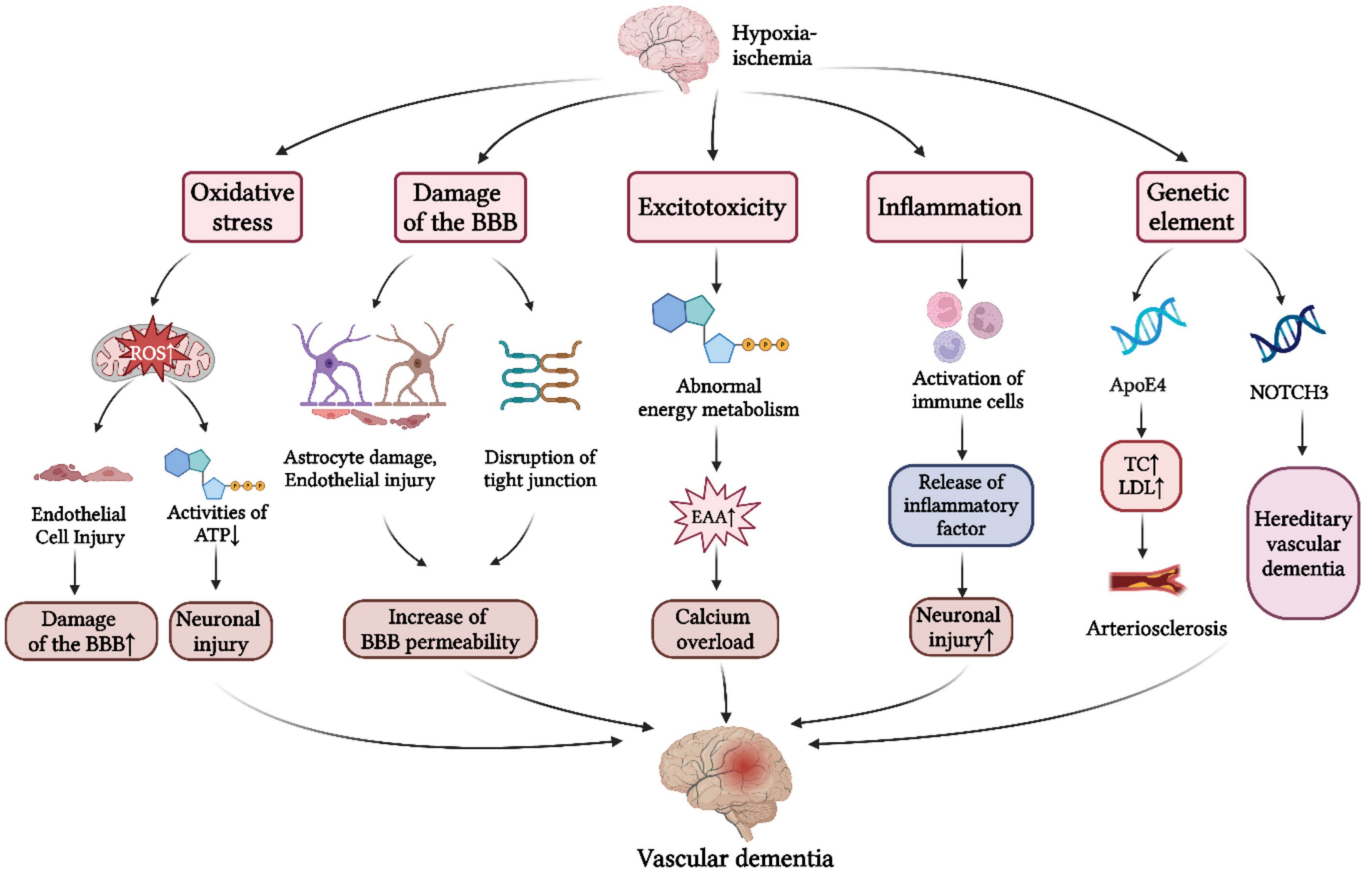
Pathogenesis of VD. The pathogenesis of VD is primarily divided into five aspects: (1) Oxidative stress response. Cerebral hypoxia-ischemia induces a marked elevation in ROS production, which contributes to the damage of BBB and neuronal injury; (2) BBB damage. Under conditions of cerebral hypoperfusion, the resultant the increased BBB permeability facilitates neurotoxic substances to enter the cerebral parenchyma, thereby precipitating neuronal injury; (3) Excitotoxic effects. Excessive release and impaired reuptake of EAAs following CIRI initiates calcium overload, ultimately contributing to the progression of VD; (4) Neuroinflammatory response. Cerebral hypoxia-ischemia triggers the release of inflammatory factors by immune cells, and subsequent inflammatory infiltration of cerebral parenchyma, ultimately culminating in neuronal dysfunction; (5) Genetic mechanisms. Apo E4 gene and the NOTCH3 gene have been identified as major genetic determinants of VD. BBB, blood brain barrier; ATP, adenosine triphosphate; EAAs, excitatory amino acids; CIRI, cerebral ischemia–reperfusion injury; TC, total cholesterol; LDL, low density lipoprotein; ApoE4, apolipoprotein E4; NOTCH3, notch receptor 3 (Created with BioRender.com).

The pathogenesis mentioned above has been summarized in Figure 1.

## 3 The role of the intestinal flora in VD

The human body is inhabited by trillions of microorganisms, including bacteria, fungi, and viruses. These symbiotic microorganisms exert a spectrum of effects on the host, which can be classified as harmful, beneficial, or neutral, and they play distinct regulatory roles in numerous physiological and pathological processes. Most of these microorganisms reside in the gastrointestinal (GI) tract, where they establish dynamic ecological communities collectively termed the intestinal flora. The gut microbiota is primarily composed of three functional groups: commensal flora—typically beneficial microbes that support host health; opportunistic pathogenic flora—normally harmless but potentially pathogenic under certain conditions; and pathogenic flora—microbes associated with disease. At the phylum level, the gut microbiota is dominated by four major groups: Firmicutes, Bacteroidetes, Proteobacteria, and Actinobacteria (Singh et al., 2022). As a vital part of the human body, these microorganisms are involved in the host’s physiological processes, such as metabolism, nutrition, and immune response (Jin et al., 2019). Furthermore, beyond their physiological roles, they also influence the host’s pathological changes, including inflammatory responses and oxidative stress, establishing a mutually beneficial symbiotic relationship with the host. Ullah et al. have demonstrated that intestinal microbiota dysbiosis may contribute to pathological activation of innate immune system, subsequently inducing a cascade of pro-inflammatory responses (Ullah et al., 2024). Table 1 shows the predominant gut microorganisms and their functions in human physiology. Moreover, gut microbiota has already shown its significant potential in treating GI diseases (Ullah et al., 2025). With the deepening of research, studies have demonstrated that there are significant alterations in the types of intestinal flora in patients with neurological disorders (Table 2), which also have been observed in patients with VD, suggesting a potential role of gut microbial imbalance in VD pathogenesis.

**Table 1:** The common gut microorganisms and their functions.

Microbiota	Beneficial	Pernicious	Functions	References
*Ruminococcus*	√		Decomposition of high fiber foods and produce substances such as butyric acid to protect the intestines	Crost et al. (2023)
*Lactobacillus*	√		Decomposition of various indigestible substances; Modulation of innate immune response	O’Callaghan and O’Toole (2011)
*Bifidobacterium*	√		Modulation of the immune system and silence intestinal inflammation early in life	Henrick et al. (2021)
*Roseburia*	√		Production of butyric acid which help to restore the integrity of the intestinal barrier	Patterson et al. (2017), Seo et al. (2020)
*Klebsiella*		√	Arousal of the metastatic infections of the lung, urinary tract, eye, and CNS	Gonzalez-Ferrer et al. (2021)
*Shigella*		√	Destruction of intestinal mucosa which cause death and ulceration of intestinal epithelial cells; And activate innate immune response that leads to primary tissue damage	Hansen et al. (2021), Lee and Tesh (2019)
*Brucella*		√	Inducement of acute or chronic infection of reproductive tract leading to miscarriage or serious reproductive tract disorders	Głowacka et al. (2018)
*Salmonella*		√	Invasion of intestinal epithelial cells, and initiate the immune system and initiate an inflammatory response	Kurtz et al. (2017)

**Table 2:** The dysbiosis of intestinal flora in patients or animals with neurological disorders.

Diseases	Patient/animal	Increase of intestinal flora	Decrease of intestinal flora	References
AD	Patient and animal	*Bacteroidetes*, *Proteobacteria*, etc.	*Firmicutes*, *Bifidobacterium*, etc.	Chandra et al. (2023)
Depression	Patient	*Actinomycetes*, *Bifidobacteria*, *Clostridium*, etc.	*Prevoodoceae*, *Bacteroideae*, *Coniophytae*, etc.	Simpson et al. (2021), Liu et al. (2023)
Schizophrenia	Patient	*Alcaliophilus*, *Anaerococcus*, *Macrococcus*, etc.	*Bifidobacterium*, *Coprococcus*, *Ruminococcaceae*, etc.	McGuinness et al. (2022), Nikolova et al. (2021)
PD	Patient	*Bifidobacterium*, *Enterococcus*, *Akkermansia*, etc.	*Lachnospiraceae*, *Prevotella*, *Faecalibacterium*, *Roseburia*, etc.	Hirayama and Ohno (2021), Li et al. (2023)
Epilepsy	Patient	*Fusobacterium*, *Megasphaera*, *Alloprevotella*, etc.	*Bifidobacteria*, etc.	Dahlin and Prast-Nielsen (2019), Dong et al. (2022)
Huntington’s disease	Patient and animal	*Actinobacteria*, *Lactobacillaceae*, *Lactobacillus*, etc.	*Prevotellaceae*, *Clostridium XVIII*, etc.	Ekwudo et al. (2024), Sharma et al. (2023)
Stroke	Patient	*Actinobacteria*, *Proteobacteria*, *Enterobacteriaceae*, etc.	*Bacteroidetes*, *Firmicutes*, *genera Faecalibacterium*, etc.	Peh et al. (2022), Tan et al. (2020)

And further research has underscored that intestinal flora is inextricably linked to the patients with VD. In a case–control study, Yin et al. observed a marked disruption in the balance of intestinal flora among VD patients, featuring elevated levels of conditional pathogenic bacteria such as *Enterobacteriaceae*, *Barnesiella intestinihominis*, *Megasphaera*, and *Osteobacteria*, alongside a diminished abundance of beneficial symbiotic bacteria, including *Bacteroides* and *Prevotella* (Yin et al., 2015). And relevant statistical data and clinical investigations further reveal that VD patients infected with *Enterobacteriaceae* (e.g., *Helicobacter pylori*) exhibit more severe cognitive deficits compared to their uninfected counterparts, potentially mediated by upregulated pro-inflammatory cytokines, such as IL-α, IL-1β, IL-6, and IL-8 (Xu et al., 2016). Furthermore, Xiao et al. observed that in chronic cerebral hypoperfusion (CCH) rats with cognitive impairment, there was a decrease in the abundance of representative short-chain fatty acids (SCFAs) producers, including members of the Prevotellaceae family and the Bifidobacterium genus (Xiao W. et al., 2022). SCFAs, as important metabolites of gut microbiota, primarily consist of acetic, propionic, and butyric acids, which influence neuronal function by modulating calcium signaling and neurotransmitter release (Xu et al., 2023). Mirzaei et al. have demonstrated that butyric acid can inhibit neuronal apoptosis and ameliorate histopathological changes in the CA1 region through the BDNF/PI3K/Akt pathway, thereby counteracting VD (Mirzaei et al., 2021). Additionally, butyrate has been shown to suppress neuroinflammation, alleviate chronic alcoholic CNS impairment, and enhance cognitive and memory functions via the GPR109A/PPAR-γ/TLR4-NF-κB signaling pathway (Wei et al., 2023). Furthermore, besides the SCFAs mentioned above, trimethylamine N-oxide (TMAO)—an intestinal microbe-dependent metabolite produced from dietary choline metabolism—has been associated with vascular cognitive disorders and cerebrovascular diseases (Tu and Xia, 2024). A study on intestinal microbiota transplantation has shown that elevated levels of TMAO can exacerbate the severity of stroke and cognitive deficits in a mouse model of cognitive impairment associated with stroke. Simultaneously, RNA sequencing analysis has demonstrated that TMAO accelerates the onset of VD by modifying the neuroinflammatory environment through the upregulation of a subset of genes associated with IL-17 signaling, including Lcn2, S100a8, and Ccl2 (Kaur et al., 2023). Furthermore, TMAO activates the NLRP3 inflammasome, which subsequently activates pro-caspase-1, thereby inducing inflammation (Olejnik and Golenia, 2024). Consequently, reducing TMAO levels in the body may serve as an adjunctive strategy for the treatment of VD, necessitating further validation through additional animal experiments and clinical studies.

In summary, alterations in intestinal flora and their metabolites (such as SCFAs and TMAO) may modulate apoptotic pathways and neuroinflammatory responses in VD via related signaling pathways, including BDNF/PI3K/Akt. These findings highlight the potential of intestinal flora and associated metabolites as promising biomarkers or novel therapeutic targets in VD.

## 4 The correlation between GBA and VD

**Figure 2:**
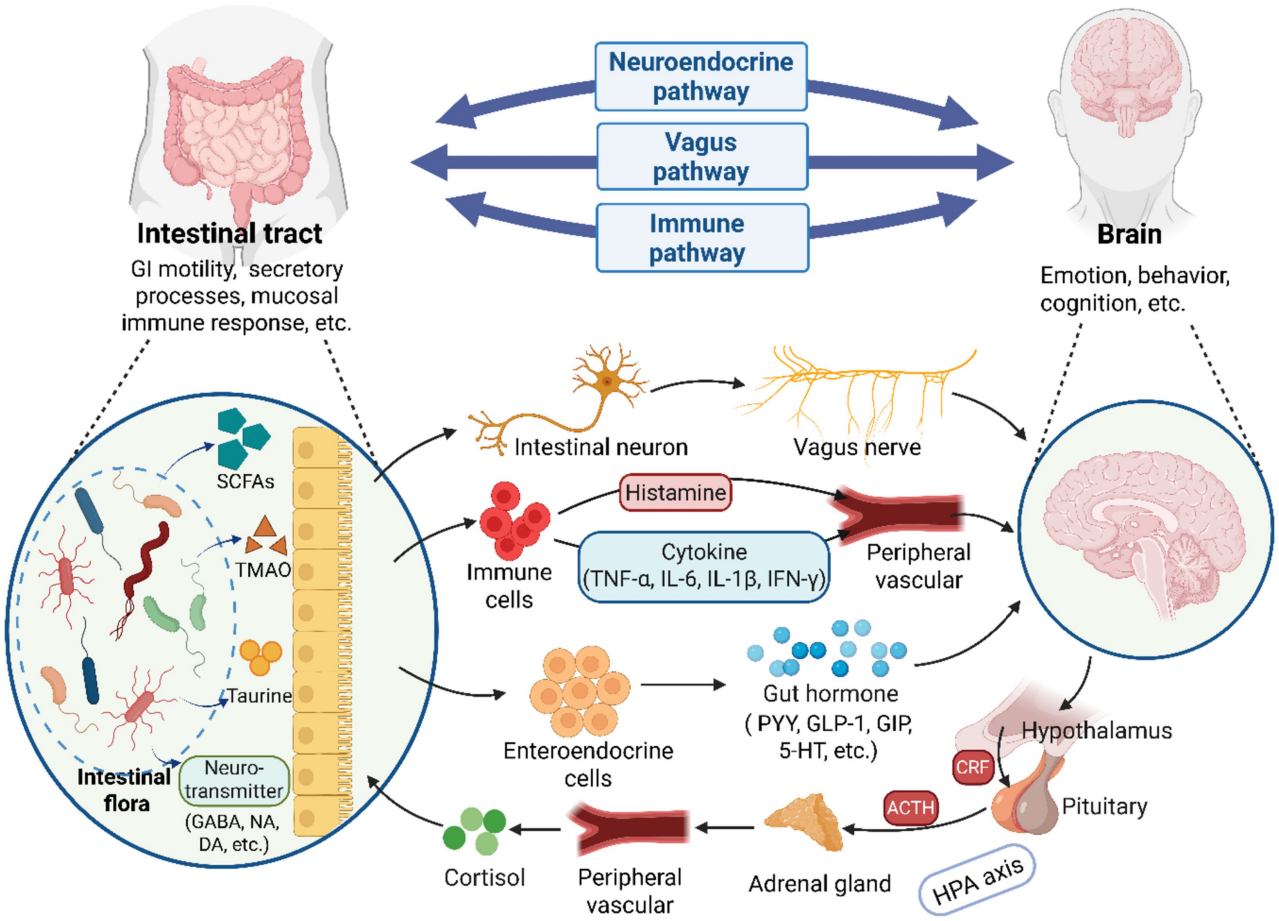
A illustration of the bidirectional communication between the brain and gut microbiota via the GBA. The GBA consists of three principal pathways: vagal nerve pathways, immune regulation pathways and neuroendocrine pathways. Microorganisms in the GI tract can establish direct neural connections with the brain by activating vagal afferents through the enteric nervous system. Additionally, gut microbial, and their synthesized metabolites and neurotransmitters regulate brain physiology by modulating immune cells to produce or release cytokines such as TNF-α. Alternatively, they may influence brain activities by stimulating enteroendocrine cells to release hormones (e.g., GABA). Furthermore, neuroendocrine hormones, particularly cortisol released via the HPA axis, play a significant role in regulating gut microbial homeostasis. GI, gastrointestinal; SCFAs, short-chain fatty acids; TMAO, trimethylamine N-oxide; GABA, γ-aminobutyric acid; NA, noradrenaline; DA, dopamine; PYY, peptideYY; GLP-1, peptide-1; GIP, gastric inhibitory polypeptide; 5-HT, serotonin; ACTH, adrenocorticotropic hormone; CRF, corticotropin-releasing factor (Created with BioRender.com).

The conventional wisdom holds that, when the CNS receives signals related to changes in both internal and external environments, it integrates various messages from different brain centers and transmits these regulatory signals to the gut. This modulation occurs either through the autonomic nervous system or the endocrine system, impacting intestinal barrier integrity, GI motility, secretory processes, and the mucosal immune response (Zhu et al., 2017; Mayer et al., 2022). However, with the advancement of research, accumulating evidence has demonstrated that the intestinal flora not only impacts GI system but also interacts with the physiological functions of the brain. This intricate crosstalk enables gut microorganisms to modulate cerebral growth, neurodevelopment, and synaptic plasticity through three principal mechanistic pathways: vagal nerve pathways, immune regulation pathways, and neuroendocrine pathways (Mayer et al., 2022). Additionally, relevant studies have indicate that gut microbiota can directly or indirectly participate in the pathogenesis and progression of various neurological disorders through its capacity to modulate neurophysiological processes in the CNS (Cryan and O’Mahony, 2011; Luna and Foster, 2015; Ullah et al., 2023). The primary pathway for this interaction is the GBA, which serves as a continuous, bidirectional communication channel between gut microbiota and the brain, encompassing the hypothalamic–pituitary–adrenal (HPA) axis, the CNS, and the ENS (Cryan et al., 2019). The hypothetical bidirectional connections regulating the GBA have been illustrated in Figure 2.

Zhao et al. found that rotenone-induced intestinal flora dysbiosis in rats leads to LPS-mediated intestinal inflammation, which results in the leakage of LPS and pro-inflammatory factors into the bloodstream due to increased intestinal permeability. This process activates the TLR4/MyD88/NF-κB signaling pathway in the BBB via the GBA, resulting in neuroinflammatory responses (Zhao et al., 2021) that further contribute to cognitive impairment (Zou et al., 2023). Acupuncture has been shown to alleviate cognitive impairment in VD rats by inhibiting this signaling pathway, thereby reducing microglial inflammation (Wang et al., 2020). Another study indicates that severe disruption of intestinal flora homeostasis can cause GBA dysfunction, leading to autonomic nervous system dysfunction and abnormal secretion of the HPA axis (Spaziani et al., 2008), significantly affecting hippocampal function (Möhle et al., 2016). For instance, in a male CCH rat model, the HPA axis is abnormally activated, resulting in sustained production of corticosterone and the development of insulin resistance, which is a significant factor accelerating the progression of dementia (Lansdell and Dorrance, 2022). Additionally, serotonin (5-HT), secreted by enterochromaffin cells (ECCs), is an important neurotransmitter synthesized from tryptophan and can influence brain function via the bloodstream or the vagus nerve (Xing et al., 2024). Clinical studies have reported that the expression levels of 5-HT are reduced in the brains of AD patients and in the plasma of Parkinson’s disease (PD) patients (Mattsson et al., 2022; de Natale et al., 2021). However, Aaldijk and his colleagues have revealed that fecal microbiota transplantation (FMT) and probiotics can enhance the vagal pathway, promoting serotonergic neurotransmission in the hippocampus, which exerts beneficial effects on AD treatment (Aaldijk and Vermeiren, 2022). Currently, 5-HT-targeted drugs in clinical trials have shown potential in improving memory and learning abilities. However, their clinical application remains limited. Notably, to date, no 5-HT-modulating agents have been approved for clinical use in the management of VD (Xing et al., 2024).

## 5 CHM therapy for VD based on intestinal flora

CHM is the core component of traditional Chinese medicine with a history of thousands of years, and functions through compound compatibility and holistic regulation (such as balancing qi, blood, yin, and yang), which aligns with the concept of “multi-target therapy” in modern medicine. CHM can be digested and absorbed through the GI tract, regulating the composition of intestinal flora and the production of metabolites. These Chinese herbals increase the diversity of intestinal flora, protect the intestinal mucosal barrier, and inhibit the intestinal inflammatory response, which collectively contribute to prevent and treat VD.

### 5.1 Active ingredients of CHM and VD

The active ingredients of CHM serve as the material basis for its efficacy, possessing clear chemical structures and specific action targets, have higher targeting specificity for treating VD. Behavioral tests such as the light–dark box test and neuronal structure observation were conducted on mice that received oral administration of berberine (BBR) for 7 days after treatment for cerebral ischemia–reperfusion injury (CIRI). The findings indicated that BBR pretreatment ameliorated cognitive behavioral deficits in CIRI mice, alleviated neuronal structural damage, and significantly increased GSH levels. Moreover, 16S rRNA results demonstrated that BBR administration elevated levels of *Bacteroidetes* and *Enterobacteriaceae* within the intestinal flora of mice experiencing CIRI. The aforementioned effects were attributed to BBR’s reduction of oxidative stress damage by modulating the gut microbiota via the GBA (Wang et al., 2023). Additionally, a randomized clinical trial has shown that BBR has achieved initial clinical translation and is now applied in the clinical treatment of cognitive deficits in patients with chronic schizophrenia (Pu et al., 2023). Zhu and his colleagues administered puerarin to rats with bilateral common carotid artery occlusion (BCCAO) via gavage at doses of 50, 100, or 150 mg/kg over a period of 5 weeks. The experimental effects of puerarin were investigated using methodologies such as the Morris water maze and neuropathological analyses, including HE staining and Golgi staining. The results indicated that puerarin treatment significantly improved spatial cognitive impairments in VD rats and notably increased the relative abundance of beneficial microorganisms as well as the content of SCFAs in the gut (Zhu et al., 2021). Cui et al. conducted a study in which PD rats received daily curcumin administration (25, 100, or 400 mg/kg) via oral gavage for four consecutive weeks, followed by intestinal flora analysis using 16S rRNA sequencing. The findings demonstrated that curcumin could alleviate motor deficits in PD rats and increase the abundance of gut microorganisms, such as *Muribaculaceae* and *Eggerthellaceae*. Subsequent FMT confirmed that the neuroprotective effects of curcumin were mediated by modulating gut microbiota metabolites through the GBA (Cui et al., 2022). To date, the majority of clinical trial data have primarily established curcumin’s therapeutic potential as an anti-inflammatory agent, showing symptomatic relief in patients with diverse inflammatory conditions (Panknin et al., 2023). There is a notable lack of clinical evidence supporting its mechanism of action through the modulation of the gut microbiota. Liu et al. developed an AD mouse model by combined administration of d-galactose and aluminum chloride. The mice were then randomized into three groups and AD mice were treated with Ginkgolide B (GB) via daily intragastric gavage for 14 consecutive days. Post-treatment, cognitive function was quantitatively assessed using behavioral tests (e.g., open field test), while intestinal flora composition was analyzed by 16S rRNA gene sequencing. The results indicated that GB increased the abundance of *Bacteroides*, *Muribaculaceae*, and *Alloprevotella* in the AD mice, regulated the imbalance of intestinal flora, and significantly alleviated cognitive dysfunction (Liu J. et al., 2021). Currently, GB injection is utilized in clinical practice, and a randomized clinical trial has demonstrated its beneficial effects in improving neurological deficits in patients with IS (Zhao et al., 2022). However, its impact on gut microbiota remains unknown. In mice with CIRI, seven-day oral administration of baicalin (100 mg/kg/day) significantly altered intestinal flora composition, as demonstrated through comprehensive phenotypic evaluations (object recognition tests, morphological staining et al.) and metagenomic analyses. Treatment resulted in gut microbial remodeling, reduced plasma TMAO levels, and improved cognitive function, including enhanced learning performance and long-term potentiation (LTP)(Liu et al., 2020). Despite these encouraging preclinical findings, there are currently few instances in which baicalein is applied clinically as a monotherapeutic agent; current therapeutic strategies predominantly employ baicalein in combination with other CHM, such as Buyang Huanwu Decoction (BYHWD).

### 5.2 Chinese medicine compounds and VD

Chinese medicine compounds have the characteristics of multiple components, multiple targets, and multiple pathways. Randomized controlled clinical trials have found that BYHWD can greatly improve the cognitive dysfunction of VD patients and shows less adverse reactions (Kim et al., 2022). Similarly, Tang et al. utilized 16S rRNA sequencing to analyze the intestinal microbiota composition of rats with middle cerebral artery occlusion (MCAO), following 7-day treatment with BYHWT. Neurological function was quantitatively assessed using the modified Neurological Severity Score (mNSS) test. And they discovered that BYHWT could alleviate inflammatory responses and ameliorate neuronal damage by significantly reducing the proportion of pro-inflammatory bacteria such as *Shigella*, *Klebsiella*, and *Streptococcus* in the gut, while simultaneously increasing the abundance of beneficial bacteria like *Lactobacillus* and *Faecalibacterium* (Tang et al., 2022). It can be inferred that BYHWD may exert neuroprotective effects by modulating the intestinal microbiota via the GBA; however, the specific regulatory mechanisms warrant further investigation. Xixin Decoction (XXD) is effective in resolving phlegm and opening the orifices. Clinically, it is primarily utilized to enhance learning and memory abilities in patients diagnosed with AD (Zhao et al., 2023). Animal experiments have demonstrated that continuous intragastric administration of XXD at a dosage of 0.94 g/kg/d for 28 days, followed by 16S rRNA sequencing, resulted in a significant increase in the abundance of *Bacteroidetes* and *Firmicutes* in the intestines of rats with AD. Additionally, the diversity of the intestinal microbiota exhibited an upward trend. Western blot analysis revealed a marked increase in the expression of brain-derived neurotrophic factor (BDNF) protein in both hippocampal and colonic tissues. Concurrently, improvements were noted in learning and memory functions, motor response capabilities, and cognitive impairments (Wang et al., 2021). In a separate study, APP/PS1 transgenic mice with dementia were orally administered Qifu Yin (QFY) for 3 months. Researchers objectively assessed the cognitive levels of these mice using the step-down test and Morris water maze test, while also analyzing intestinal flora through 16S rRNA amplicon sequencing and metagenomic sequencing. The findings indicated that QFY could enhance the structure of the gut microbiota in mice, regulate the species richness of *Bacteroidetes* and *Erysipelotrichia*, and thereby ameliorate cognitive dysfunction (Xiao Q. Y. et al., 2022). However, the current clinical focus predominantly emphasizes the cognitive improvement effects of QFY and XXD, and has yet to establish whether the observed cognitive improvements are mediated by gut microbiota modulation, as this potential mechanistic pathway remains under investigation in the existing literature. Zhang et al. administered Tong-Qiao-Huo-Xue Decoction (TQHXD) via gavage to CIRI rat models established using MCAO for 7 and 14 days, subsequently conducting high-throughput 16S rDNA sequencing. They found that the proportion of *Bacteroidetes* in the intestines of CIRI rats increased compared to the model group, reversing the reduction of *Bifidobacterium* and *Lactobacillus*, thereby protecting the integrity of the intestinal epithelial mucosa (Zhang et al., 2020). Meanwhile, the inflammatory response was suppressed, which is a significant factor in the development of VD.

In summary, it is evident that CHM can enhance cognitive functions in neurological disorder such as VD by modulating the intestinal flora. However, current research primarily focuses on isolated studies of either the brain or the gut, with limited investigations into the GBA. Consequently, there is a pressing need for more fundamental research in this area, which presents significant potential for exploration.

## 6 Summary

Many current studies indicate that neuronal damage, inflammatory responses, and other factors are associated with the pathogenesis of VD, while intestinal flora has been proven to have the ability to maintain immunity, inhibit neuroinflammation, and repair neuronal damage. For example, multiple studies have shown that after microbiota transplantation, the transplanted probiotics can maintain the homeostasis of intestinal flora, reduce the occurrence of inflammatory reactions, and improve cognitive abilities. It is speculated that the intestinal flora participates in the bidirectional regulation of the GBA through immunity, endocrine or vagus nerve, and plays an important role in the cognitive activities of VD patients. The correlation and the underlying mechanisms involving intestinal flora and VD are still in the theoretical discussion stage, as they currently lack sufficient backing from both experimental and clinical research data. Therefore, it is of great research significance to use intestinal flora as a target to explore the mechanism of improving VD patients through the GBA. Further confirmation is needed to determine whether CHM can inhibit neuroinflammation, stabilize immunity and other functions by improving intestinal flora. And it is crucial to conduct in-depth research on the mechanisms of action of CHM to offer more definitive evidence for clinical management of VD.

## References

[ref1] Aaldijk E. Vermeiren Y. (2022). The role of serotonin within the microbiota-gut-brain axis in the development of Alzheimer’s disease: a narrative review. Ageing Res. Rev. 75:101556. doi: 10.1016/j.arr.2021.101556, PMID: 34990844

[ref2] Akgül G. McBain C. J. (2016). Diverse roles for ionotropic glutamate receptors on inhibitory interneurons in developing and adult brain. J. Physiol. 594, 5471–5490. doi: 10.1113/JP271764, PMID: 26918438 PMC5043048

[ref3] Carvalho C. Moreira P. I. (2018). Oxidative stress: A major player in cerebrovascular alterations associated to neurodegenerative events. Front. Physiol. 9:806. doi: 10.3389/fphys.2018.00806, PMID: 30018565 PMC6037979

[ref4] Chandra S. Sisodia S. S. Vassar R. J. (2023). The gut microbiome in Alzheimer’s disease: what we know and what remains to be explored. Mol. Neurodegener. 18:9. doi: 10.1186/s13024-023-00595-7, PMID: 36721148 PMC9889249

[ref5] Cho B. P. H. Harshfield E. L. Al-Thani M. Tozer D. J. Bell S. Markus H. S. (2022). Association of vascular risk factors and genetic factors with penetrance of variants causing monogenic stroke. JAMA Neurol. 79, 1303–1311. doi: 10.1001/jamaneurol.2022.3832, PMID: 36300346 PMC9614680

[ref6] Crost E. H. Coletto E. Bell A. Juge N. (2023). *Ruminococcus gnavus*: friend or foe for human health. FEMS Microbiol. Rev. 47:fuad014. doi: 10.1093/femsre/fuad014, PMID: 37015876 PMC10112845

[ref7] Cryan J. F. O’Mahony S. M. (2011). The microbiome-gut-brain axis: from bowel to behavior: from bowel to behavior. Neurogastroenterol. Motil. 23, 187–192. doi: 10.1111/j.1365-2982.2010.01664.x, PMID: 21303428

[ref8] Cryan J. F. O’Riordan K. J. Cowan C. S. M. Sandhu K. V. Bastiaanssen T. F. S. Boehme M.. (2019). The microbiota-gut-brain axis. Physiol. Rev. 99, 1877–2013. doi: 10.1152/physrev.00018.2018 31460832

[ref9] Cui C. Han Y. Li H. Yu H. Zhang B. Li G. (2022). Curcumin-driven reprogramming of the gut microbiota and metabolome ameliorates motor deficits and neuroinflammation in a mouse model of Parkinson’s disease. Front. Cell. Infect. Microbiol. 12:887407. doi: 10.3389/fcimb.2022.887407, PMID: 36034698 PMC9400544

[ref10] Dahlin M. Prast-Nielsen S. (2019). The gut microbiome and epilepsy. EBioMedicine 44, 741–746. doi: 10.1016/j.ebiom.2019.05.024 31160269 PMC6604367

[ref11] Davidson Y. Gibbons L. Purandare N. Byrne J. Hardicre J. Wren J.. (2006). Apolipoprotein E υ4 allele frequency in vascular dementia. Dement. Geriatr. Cogn. Disord. 22, 15–19. doi: 10.1159/000092960, PMID: 16645276

[ref12] de Natale E. R. Wilson H. Politis M. (2021). Serotonergic imaging in parkinson’s disease. Prog. Brain Res. 261, 303–338. doi: 10.1016/bs.pbr.2020.11.001, PMID: 33785134

[ref13] Dong L. Zheng Q. Cheng Y. Zhou M. Wang M. Xu J.. (2022). Gut microbial characteristics of adult patients with epilepsy. Front. Neurosci. 16:803538. doi: 10.3389/fnins.2022.803538, PMID: 35250450 PMC8888681

[ref14] Ekwudo M. N. Gubert C. Hannan A. J. (2024). The microbiota-gut-brain axis in Huntington’s disease: pathogenic mechanisms and therapeutic targets. FEBS J. 292, 1282–1315. doi: 10.1111/febs.17102, PMID: 38426291 PMC11927060

[ref15] Głowacka P. Żakowska D. Naylor K. Niemcewicz M. Bielawska-Drózd A. (2018). *Brucella* – virulence factors, pathogenesis and treatment. Pol. J. Microbiol. 67, 151–161. doi: 10.21307/pjm-2018-029, PMID: 30015453 PMC7256693

[ref16] Gonzalez-Ferrer S. Peñaloza H. F. Budnick J. A. Bain W. G. Nordstrom H. R. Lee J. S.. (2021). Finding order in the chaos: outstanding questions in *Klebsiella pneumoniae* pathogenesis. Infect. Immun. 89, e00693–e00620. doi: 10.1128/IAI.00693-20, PMID: 33558323 PMC8090965

[ref17] Guo L. Jiao B. Liao X. Xiao X. Zhang W. Yuan Z.. (2021). The role of *NOTCH3* variants in Alzheimer’s disease and subcortical vascular dementia in the Chinese population. CNS Neurosci. Ther. 27, 930–940. doi: 10.1111/cns.13647, PMID: 33942994 PMC8265940

[ref18] Hansen J. M. De Jong M. F. Wu Q. Zhang L.-S. Heisler D. B. Alto L. T.. (2021). Pathogenic ubiquitination of GSDMB inhibits NK cell bactericidal functions. Cell 184, 3178–3191.e18. doi: 10.1016/j.cell.2021.04.036, PMID: 34022140 PMC8221529

[ref19] Henrick B. M. Rodriguez L. Lakshmikanth T. Pou C. Henckel E. Arzoomand A.. (2021). Bifidobacteria-mediated immune system imprinting early in life. Cell 184, 3884–3898.e11. doi: 10.1016/j.cell.2021.05.030, PMID: 34143954

[ref20] Hirayama M. Ohno K. (2021). Parkinson’s disease and gut microbiota. Ann. Nutr. Metab. 77, 28–35. doi: 10.1159/000518147, PMID: 34500451

[ref21] Jin M. Qian Z. Yin J. Xu W. Zhou X. (2019). The role of intestinal microbiota in cardiovascular disease. J. Cell. Mol. Med. 23, 2343–2350. doi: 10.1111/jcmm.14195, PMID: 30712327 PMC6433673

[ref22] Jurcau A. Simion A. (2021). Neuroinflammation in cerebral ischemia and ischemia/reperfusion injuries: from pathophysiology to therapeutic strategies. IJMS 23:14. doi: 10.3390/ijms23010014, PMID: 35008440 PMC8744548

[ref23] Kaur N. LaForce G. Mallela D. P. Saha P. P. Buffa J. Li X. S.. (2023). Exploratory transcriptomic profiling reveals the role of gut microbiota in vascular dementia. Int. J. Mol. Sci. 24:8091. doi: 10.3390/ijms24098091, PMID: 37175797 PMC10178712

[ref24] Kesika P. Suganthy N. Sivamaruthi B. S. Chaiyasut C. (2021). Role of gut-brain axis, gut microbial composition, and probiotic intervention in Alzheimer’s disease. Life Sci. 264:118627. doi: 10.1016/j.lfs.2020.118627, PMID: 33169684

[ref25] Kim D. W. Kim S. H. Kook H. J. Jung I. C. (2022). Efficacy and safety of Buyang-Huanwu-Tang (Boyang-Hwano-Tang) in patients with vascular dementia: A systematic review and meta-analysis. Complement. Ther. Clin. Pract. 47:101547. doi: 10.1016/j.ctcp.2022.101547, PMID: 35168040

[ref26] Kurtz J. R. Goggins J. A. McLachlan J. B. (2017). Salmonella infection: interplay between the bacteria and host immune system. Immunol. Lett. 190, 42–50. doi: 10.1016/j.imlet.2017.07.006, PMID: 28720334 PMC5918639

[ref27] Lansdell T. A. Dorrance A. M. (2022). Chronic cerebral hypoperfusion in male rats results in sustained HPA activation and hyperinsulinemia. Am. J. Physiol. Endocrinol. Metab. 322, E24–E33. doi: 10.1152/ajpendo.00233.2021, PMID: 34747203 PMC8721904

[ref28] Lee M.-S. Tesh V. L. (2019). Roles of Shiga toxins in immunopathology. Toxins 11:212. doi: 10.3390/toxins11040212, PMID: 30970547 PMC6521259

[ref29] Li T. Li S. Xiong Y. Li X. Ma C. Guan Z.. (2024). Binary Nano-inhalant formulation of icariin enhances cognitive function in vascular dementia via BDNF/TrkB signaling and anti-inflammatory effects. Neurochem. Res. 49, 1720–1734. doi: 10.1007/s11064-024-04129-5, PMID: 38520637

[ref30] Li Z. Liang H. Hu Y. Lu L. Zheng C. Fan Y.. (2023). Gut bacterial profiles in Parkinson’s disease: A systematic review. CNS Neurosci. Ther. 29, 140–157. doi: 10.1111/cns.13990, PMID: 36284437 PMC9804059

[ref31] Liu S. Pu G. Li Y. Zhang H. (2021). Efficacy of Shenma Yizhi prescription combined with Citalo efficacy of Shenma Yizhi prescription combined with citalopram in the treatment with cognitive dysfunction and its effect on inflammatory factors and oxidative stress. Pract. J. Cardiac Cereb. Pneumal Vasc. Dis. 29, 77–81. doi: 10.12114/j.issn.1008-5971.2021.00.106

[ref32] Liu L. Wang H. Chen X. Zhang Y. Zhang H. Xie P. (2023). Gut microbiota and its metabolites in depression: from pathogenesis to treatment. EBioMedicine 90:104527. doi: 10.1016/j.ebiom.2023.104527, PMID: 36963238 PMC10051028

[ref33] Liu J. Ye T. Zhang Y. Zhang R. Kong Y. Zhang Y.. (2021). Protective effect of Ginkgolide B against cognitive impairment in mice via regulation of gut microbiota. J. Agric. Food Chem. 69, 12230–12240. doi: 10.1021/acs.jafc.1c05038, PMID: 34633804

[ref34] Liu J. Zhang T. Wang Y. Si C. Wang X. Wang R.-T.. (2020). Baicalin ameliorates neuropathology in repeated cerebral ischemia-reperfusion injury model mice by remodeling the gut microbiota. Aging (Albany NY) 12, 3791–3806. doi: 10.18632/aging.102846, PMID: 32084011 PMC7066900

[ref35] Luna R. A. Foster J. A. (2015). Gut brain axis: diet microbiota interactions and implications for modulation of anxiety and depression. Curr. Opin. Biotechnol. 32, 35–41. doi: 10.1016/j.copbio.2014.10.007, PMID: 25448230

[ref36] Mattsson P. Cselényi Z. Andrée B. Borg J. Nag S. Halldin C.. (2022). Decreased 5-HT1A binding in mild Alzheimer’s disease-A positron emission tomography study. Synapse 76:e22235. doi: 10.1002/syn.22235, PMID: 35587913 PMC9285435

[ref37] Mayer E. A. Nance K. Chen S. (2022). The gut–brain Axis. Annu. Rev. Med. 73, 439–453. doi: 10.1146/annurev-med-042320-014032 34669431

[ref38] McGuinness A. J. Davis J. A. Dawson S. L. Loughman A. Collier F. O’Hely M.. (2022). A systematic review of gut microbiota composition in observational studies of major depressive disorder, bipolar disorder and schizophrenia. Mol. Psychiatry 27, 1920–1935. doi: 10.1038/s41380-022-01456-3, PMID: 35194166 PMC9126816

[ref39] Mirzaei R. Bouzari B. Hosseini-Fard S. R. Mazaheri M. Ahmadyousefi Y. Abdi M.. (2021). Role of microbiota-derived short-chain fatty acids in nervous system disorders. Biomed. Pharmacother. 139:111661. doi: 10.1016/j.biopha.2021.111661 34243604

[ref40] Möhle L. Mattei D. Heimesaat M. M. Bereswill S. Fischer A. Alutis M.. (2016). Ly6C(hi) monocytes provide a link between antibiotic-induced changes in gut microbiota and adult hippocampal neurogenesis. Cell Rep. 15, 1945–1956. doi: 10.1016/j.celrep.2016.04.074, PMID: 27210745

[ref41] Nikolova V. L. Smith M. R. B. Hall L. J. Cleare A. J. Stone J. M. Young A. H. (2021). Perturbations in gut microbiota composition in psychiatric disorders: a review and meta-analysis. JAMA Psychiatry 78, 1343–1354. doi: 10.1001/jamapsychiatry.2021.2573, PMID: 34524405 PMC8444066

[ref42] O’Callaghan J. O’Toole P. W. (2011). “Lactobacillus: host–microbe relationships” in Between pathogenicity and commensalism. eds. Dobrindt U. Hacker J. H. Svanborg C. (Berlin, Heidelberg: Springer Berlin Heidelberg), 119–154. doi: 10.1007/82_2011_187

[ref43] Olejnik P. Golenia A. (2024). Vascular cognitive impairment-the molecular basis and potential influence of the gut microbiota on the pathological process. Cells 13:1962. doi: 10.3390/cells13231962, PMID: 39682711 PMC11639845

[ref44] Panknin T. M. Howe C. L. Hauer M. Bucchireddigari B. Rossi A. M. Funk J. L. (2023). Curcumin supplementation and human disease: a scoping review of clinical trials. IJMS 24:4476. doi: 10.3390/ijms24054476, PMID: 36901908 PMC10003109

[ref45] Patterson A. M. Mulder I. E. Travis A. J. Lan A. Cerf-Bensussan N. Gaboriau-Routhiau V.. (2017). Human gut symbiont *Roseburia hominis* promotes and regulates innate immunity. Front. Immunol. 8:1166. doi: 10.3389/fimmu.2017.01166, PMID: 29018440 PMC5622956

[ref46] Peh A. O’Donnell J. A. Broughton B. R. S. Marques F. Z. (2022). Gut microbiota and their metabolites in stroke: A double-edged sword. Stroke 53, 1788–1801. doi: 10.1161/STROKEAHA.121.036800, PMID: 35135325

[ref47] Pu Z. Wen H. Jiang H. Hou Q. Yan H. (2023). Berberine improves negative symptoms and cognitive function in patients with chronic schizophrenia via anti-inflammatory effect: a randomized clinical trial. Chin. Med. 18:41. doi: 10.1186/s13020-023-00746-4, PMID: 37069570 PMC10108529

[ref48] Qi D.-H. Ma H. Chen Y.-Y. Wang K.-X. Ding M.-M. Hao Y.-L.. (2023). Research progress in mechanism of puerarin in treating vascular dementia. Zhongguo Zhong Yao Za Zhi 48, 5993–6002. doi: 10.19540/j.cnki.cjcmm.20230718.401, PMID: 38114205

[ref49] Radak D. Katsiki N. Resanovic I. Jovanovic A. Sudar-Milovanovic E. Zafirovic S.. (2017). Apoptosis and acute brain ischemia in ischemic stroke. CVP 15, 115–122. doi: 10.2174/1570161115666161104095522, PMID: 27823556

[ref50] Rajeev V. Fann D. Y. Dinh Q. N. Kim H. A. De Silva T. M. Jo D.-G.. (2022). Intermittent fasting attenuates Hallmark vascular and neuronal pathologies in a mouse model of vascular cognitive impairment. Int. J. Biol. Sci. 18, 6052–6067. doi: 10.7150/ijbs.75188, PMID: 36439869 PMC9682544

[ref51] Seo B. Jeon K. Moon S. Lee K. Kim W.-K. Jeong H.. (2020). Roseburia spp. abundance associates with alcohol consumption in humans and its administration ameliorates alcoholic fatty liver in mice. Cell Host Microbe 27, 25–40.e6. doi: 10.1016/j.chom.2019.11.001, PMID: 31866426

[ref52] Shan L. Fan Y. Yuanchun C. Ruoxi Z. Haiye L. Fei G.. (2024). Sanhua Tang protects against ischemic stroke by preventing blood-brain barrier injury: a network pharmacology and experiments. J. Tradit. Chin. Med. 44, 794–803. doi: 10.19852/j.cnki.jtcm.20240515.001, PMID: 39066540 PMC11337263

[ref53] Sharma G. Biswas S. S. Mishra J. Navik U. Kandimalla R. Reddy P. H.. (2023). Gut microbiota dysbiosis and Huntington’s disease: exploring the gut-brain axis and novel microbiota-based interventions. Life Sci. 328:121882. doi: 10.1016/j.lfs.2023.121882, PMID: 37356750

[ref54] Simpson C. A. Diaz-Arteche C. Eliby D. Schwartz O. S. Simmons J. G. Cowan C. S. M. (2021). The gut microbiota in anxiety and depression - A systematic review. Clin. Psychol. Rev. 83:101943. doi: 10.1016/j.cpr.2020.101943, PMID: 33271426

[ref55] Singh S. Sharma P. Pal N. Kumawat M. Shubham S. Sarma D. K.. (2022). Impact of environmental pollutants on gut microbiome and mental health via the gut-brain Axis. Microorganisms 10:1457. doi: 10.3390/microorganisms10071457, PMID: 35889175 PMC9317668

[ref56] Smith E. E. (2017). Clinical presentations and epidemiology of vascular dementia. Clin. Sci. (Lond.) 131, 1059–1068. doi: 10.1042/CS20160607, PMID: 28515342

[ref57] Song J. Li M. Kang N. Jin W. Xiao Y. Li Z.. (2024). Baicalein ameliorates cognitive impairment of vascular dementia rats via suppressing neuroinflammation and regulating intestinal microbiota. Brain Res. Bull. 208:110888. doi: 10.1016/j.brainresbull.2024.110888, PMID: 38295883

[ref58] Spaziani R. Bayati A. Redmond K. Bajaj H. Mazzadi S. Bienenstock J.. (2008). Vagal dysfunction in irritable bowel syndrome assessed by rectal distension and baroreceptor sensitivity. Neurogastroenterol. Motil. 20, 336–342. doi: 10.1111/j.1365-2982.2007.01042.x, PMID: 18179607

[ref59] Sun C. Liu M. Liu J. Zhang T. Zhang L. Li H.. (2021). ShenmaYizhi decoction improves the mitochondrial structure in the brain and ameliorates cognitive impairment in VCI rats via the AMPK/UCP2 signaling pathway. Neuropsychiatr. Dis. Treat. 17, 1937–1951. doi: 10.2147/NDT.S302355, PMID: 34168453 PMC8218872

[ref60] Tan B. Y. Q. Paliwal P. R. Sharma V. K. (2020). Gut microbiota and stroke. Ann. Indian Acad. Neurol. 23, 155–158. doi: 10.4103/aian.AIAN_483_19, PMID: 32189854 PMC7061503

[ref61] Tang R. Yi J. Lu S. Chen B. Liu B. (2022). Therapeutic effect of Buyang Huanwu decoction on the gut microbiota and hippocampal metabolism in a rat model of cerebral ischemia. Front. Cell. Infect. Microbiol. 12:873096. doi: 10.3389/fcimb.2022.873096, PMID: 35774407 PMC9237419

[ref62] Tian D. Gao Q. Chang Z. Lin J. Ma D. Han Z. (2022). Network pharmacology and *in vitro* studies reveal the pharmacological effects and molecular mechanisms of Shenzhi Jiannao prescription against vascular dementia. BMC Complement. Med. Ther. 22:33. doi: 10.1186/s12906-021-03465-1, PMID: 35109845 PMC8812053

[ref63] Tian Z. Ji X. Liu J. (2022). Neuroinflammation in vascular cognitive impairment and dementia: current evidence, advances, and prospects. IJMS 23:6224. doi: 10.3390/ijms23116224, PMID: 35682903 PMC9181710

[ref64] Traynelis S. F. Wollmuth L. P. McBain C. J. Menniti F. S. Vance K. M. Ogden K. K.. (2010). Glutamate receptor ion channels: structure, regulation, and function. Pharmacol. Rev. 62, 405–496. doi: 10.1124/pr.109.002451, PMID: 20716669 PMC2964903

[ref65] Tu Y.-C. Kuo C.-C. (2015). The differential contribution of GluN1 and GluN2 to the gating operation of the NMDA receptor channel. Pflugers Arch. - Eur. J. Physiol. 467, 1899–1917. doi: 10.1007/s00424-014-1630-z, PMID: 25339225

[ref66] Tu R. Xia J. (2024). Stroke and vascular cognitive impairment: the role of intestinal microbiota metabolite TMAO. CNS Neurol. Disord. Drug Targets 23, 102–121. doi: 10.2174/1871527322666230203140805, PMID: 36740795

[ref67] Ullah H. Arbab S. Chang C. Bibi S. Muhammad N. Rehman S. U.. (2025). Gut microbiota therapy in gastrointestinal diseases. Front. Cell Dev. Biol. 13:1514636. doi: 10.3389/fcell.2025.1514636, PMID: 40078367 PMC11897527

[ref68] Ullah H. Arbab S. Tian Y. Chen Y. Liu C.-Q. Li Q.. (2024). Crosstalk between gut microbiota and host immune system and its response to traumatic injury. Front. Immunol. 15:1413485. doi: 10.3389/fimmu.2024.1413485, PMID: 39144142 PMC11321976

[ref69] Ullah H. Arbab S. Tian Y. Liu C.-Q. Chen Y. Qijie L.. (2023). The gut microbiota-brain axis in neurological disorder. Front. Neurosci. 17:1225875. doi: 10.3389/fnins.2023.1225875, PMID: 37600019 PMC10436500

[ref9001] Wang D. DIWU Y. Guo Y. Zhang H. Zhu X. Wang W.. (2021). Effects of Xixin Decoction on the Expression of BDNF and TrkB Protein in Hippocampus and the Diversity of Intestinal Flora of Alzheimer’s Disease Model Rats. Journal of Traditional Chinese Medicine 62, 1362–1369. doi: 10.13288/j.11-2166/r.2021.15.015

[ref70] Wang L. Yang J.-W. Lin L.-T. Huang J. Wang X.-R. Su X.-T.. (2020). Acupuncture attenuates inflammation in microglia of vascular dementia rats by inhibiting miR-93-mediated TLR4/MyD88/NF-κB signaling pathway. Oxidative Med. Cell. Longev. 2020, 8253904–8253915. doi: 10.1155/2020/8253904, PMID: 32850002 PMC7441436

[ref71] Wang X. Zhang J. Wang S. Song Z. Sun H. Wu F.. (2023). Berberine modulates gut microbiota to attenuate cerebral ferroptosis induced by ischemia-reperfusion in mice. Eur. J. Pharmacol. 953:175782. doi: 10.1016/j.ejphar.2023.175782, PMID: 37245860

[ref72] Wei H. Yu C. Zhang C. Ren Y. Guo L. Wang T.. (2023). Butyrate ameliorates chronic alcoholic central nervous damage by suppressing microglia-mediated neuroinflammation and modulating the microbiome-gut-brain axis. Biomed. Pharmacother. 160:114308. doi: 10.1016/j.biopha.2023.114308, PMID: 36709599

[ref73] Xiao W. Su J. Gao X. Yang H. Weng R. Ni W.. (2022). The microbiota-gut-brain axis participates in chronic cerebral hypoperfusion by disrupting the metabolism of short-chain fatty acids. Microbiome 10:62. doi: 10.1186/s40168-022-01255-6, PMID: 35430804 PMC9013454

[ref74] Xiao Q.-Y. Ye T.-Y. Wang X.-L. Qi D.-M. Cheng X.-R. (2022). Effects of Qi-Fu-Yin on aging of APP/PS1 transgenic mice by regulating the intestinal microbiome. Front. Cell. Infect. Microbiol. 12:1048513. doi: 10.3389/fcimb.2022.1048513, PMID: 36710967 PMC9880330

[ref75] Xing C. Chen H. Bi W. Lei T. Hang Z. Du H. (2024). Targeting 5-HT is a potential therapeutic strategy for neurodegenerative diseases. IJMS 25:13446. doi: 10.3390/ijms252413446, PMID: 39769209 PMC11679250

[ref76] Xu S. Liu Y. Wang Q. Liu F. Xian Y. Xu F.. (2023). Gut microbiota in combination with blood metabolites reveals characteristics of the disease cluster of coronary artery disease and cognitive impairment: a Mendelian randomization study. Front. Immunol. 14:1308002. doi: 10.3389/fimmu.2023.1308002, PMID: 38288114 PMC10822940

[ref77] Xu Y. Wang Q. Liu Y. Cui R. Zhao Y. (2016). Is *Helicobacter pylori* infection a critical risk factor for vascular dementia? Int. J. Neurosci. 126, 899–903. doi: 10.3109/00207454.2015.1081387, PMID: 26269142

[ref78] Yin J. Liao S.-X. He Y. Wang S. Xia G.-H. Liu F.-T.. (2015). Dysbiosis of gut microbiota with reduced trimethylamine-N-oxide level in patients with large-artery atherosclerotic stroke or transient ischemic attack. J. Am. Heart Assoc. 4:e002699. doi: 10.1161/JAHA.115.002699, PMID: 26597155 PMC4845212

[ref79] Yu W. Wang Y. (2020). Epidemiology of vascular cognitive impairment in Asia. Chinese J. Front. Med. Sci. (Electronic Version) 12, 1–8. doi: 10.12037/YXQY.2020.10-01

[ref80] Zhang Y. Shen L. Xie J. Li L. Xi W. Li B.. (2023). Pushen capsule treatment promotes functional recovery after ischemic stroke. Phytomedicine 111:154664. doi: 10.1016/j.phymed.2023.154664, PMID: 36682301

[ref81] Zhang F. Zhai M. Wu Q. Jia X. Wang Y. Wang N. (2020). Protective effect of Tong-Qiao-Huo-Xue decoction on inflammatory injury caused by intestinal microbial disorders in stroke rats. Biol. Pharm. Bull. 43, 788–800. doi: 10.1248/bpb.b19-00847, PMID: 32132347

[ref82] Zhao E.-L. Diwu Y.-C. Zhang H. Duan L.-Q. Han X.-Y. Wang Y.-L.. (2023). Xixin decoction improves learning and memory ability of SAMP8 by enhancing neuroprotective effect and inhibiting neuroinflammation. Zhongguo Zhong Yao Za Zhi 48, 5032–5040. doi: 10.19540/j.cnki.cjcmm.20230419.406 37802845

[ref83] Zhao H. Guo Q. Li B. Shi M. (2022). The efficacy and safety of Ginkgo terpene lactone preparations in the treatment of ischemic stroke: A systematic review and Meta-analysis of randomized clinical trials. Front. Pharmacol. 13:821937. doi: 10.3389/fphar.2022.821937, PMID: 35392576 PMC8982077

[ref84] Zhao Z. Ning J. Bao X.-Q. Shang M. Ma J. Li G.. (2021). Fecal microbiota transplantation protects rotenone-induced Parkinson’s disease mice via suppressing inflammation mediated by the lipopolysaccharide-TLR4 signaling pathway through the microbiota-gut-brain axis. Microbiome 9:226. doi: 10.1186/s40168-021-01107-9, PMID: 34784980 PMC8597301

[ref85] Zhu X. Han Y. Du J. Liu R. Jin K. Yi W. (2017). Microbiota-gut-brain axis and the central nervous system. Oncotarget 8, 53829–53838. doi: 10.18632/oncotarget.17754, PMID: 28881854 PMC5581153

[ref86] Zhu T. Zhu M. Qiu Y. Wu Z. Huang N. Wan G.. (2021). Puerarin alleviates vascular cognitive impairment in vascular dementia rats. Front. Behav. Neurosci. 15:717008. doi: 10.3389/fnbeh.2021.717008, PMID: 34720898 PMC8554240

[ref87] Zou H. Chen X. Lu J. Zhou W. Zou X. Wu H.. (2023). Neurotropin alleviates cognitive impairment by inhibiting TLR4/MyD88/NF-κB inflammation signaling pathway in mice with vascular dementia. Neurochem. Int. 171:105625. doi: 10.1016/j.neuint.2023.105625, PMID: 37774797

